# Quantitative Chemotherapeutic Profiling of Gynecologic Cancer Cell Lines Using Approved Drugs and Bioactive Compounds^1^

**DOI:** 10.1016/j.tranon.2018.11.016

**Published:** 2018-12-18

**Authors:** Kirill Gorshkov, Ni Sima, Wei Sun, Billy Lu, Wei Huang, Jameson Travers, Carleen Klumpp-Thomas, Samuel G. Michael, Tuan Xu, Ruili Huang, Emily M. Lee, Xiaodong Cheng, Wei Zheng

**Affiliations:** * National Center for Advancing Translational Sciences, National Institutes of Health, 9800 Medical Center Drive, Bethesda, MD 20892, USA; † Department of Gynecologic Oncology, Women's Reproductive Health Laboratory of Zhejiang Province, Women's Hospital, School of Medicine, Zhejiang University, 866 Yuhangtang Rd, Hangzhou 310058, PR China

## Abstract

Heterogeneous response to chemotherapy is a major issue for the treatment of cancer. For most gynecologic cancers including ovarian, cervical, and placental, the list of available small molecule therapies is relatively small compared to options for other cancers. While overall cancer mortality rates have decreased in the United States as early diagnoses and cancer therapies have become more effective, ovarian cancer still has low survival rates due to the lack of effective treatment options, drug resistance, and late diagnosis. To understand chemotherapeutic diversity in gynecologic cancers, we have screened 7914 approved drugs and bioactive compounds in 11 gynecologic cancer cell lines to profile their chemotherapeutic sensitivity. We identified two HDAC inhibitors, mocetinostat and entinostat, as pan-gynecologic cancer suppressors with IC_50_ values within an order of magnitude of their human plasma concentrations. In addition, many active compounds identified, including the non-anticancer drugs and other compounds, diversely inhibited the growth of three gynecologic cancer cell groups and individual cancer cell lines. These newly identified compounds are valuable for further studies of new therapeutics development, synergistic drug combinations, and new target identification for gynecologic cancers. The results also provide a rationale for the personalized chemotherapeutic testing of anticancer drugs in treatment of gynecologic cancer.

## Introduction

The five main gynecologic cancers, including ovarian, cervical, uterine, vaginal, and vulvar, correspond to 12% (94,990) of new female cancer diagnoses annually in the United States [1]. Of those, uterine endometrial, ovarian, and cervical are the most prevalent, with ovarian being the fifth leading cause of death from cancer for females in the United States [2]. In 2018, it is estimated that there will be 22,240 new ovarian cancer cases (2.5% of all female cancer cases) and 14,070 ovarian cancer deaths (5% of all female cancer deaths) [2]. The high case-to-fatality ratio exhibited in ovarian cancer can be attributed to late-stage diagnosis, lack of effective drug therapies, and tumor heterogeneity. Thus, it is important to discover new therapeutics for ovarian cancers that can improve survival in late-stage ovarian cancer patients.

While ovarian cancer is usually diagnosed at later stages of disease, resulting in a low 5-year survival of 29% for distant-stage disease, cervical cancer is typically diagnosed at early stages and thus has more favorable outcomes [2]. However, in 2017, it was found that cervical cancer death rates have been underestimated due to the prior inclusion of women who have had hysterectomies [3]. Additionally, and importantly, this study identified a large disparity in race, where black women were dying at a 77% higher rate (10.1 in 100,000 vs. 5.4 in 100,000) while white women were dying at a 47% (4.7 in 100,000 vs. 3.2 in 100,000) higher rate than previously calculated without the hysterectomy exclusion criteria. Thus, cervical cancer remains a critical driver of mortality in women.

Placental cancers, or gestational trophoblastic disease (GTD) choriocarcinomas, are another type of gynecologic cancer. Gestational carcinomas arise from the fetal-derived layer of cells called the trophoblast that surrounds an embryo [4] and are rare, with an incidence ranging from approximately 1 in 15,000 to 50,000 [4], [5]. A combination of surgery, radiation, and chemotherapy is the common treatment modality for gynecologic cancers [6].

There is currently a set of standard anticancer drugs used in the clinic to treat gynecologic cancers. For ovarian and cervical cancer, these include chemotherapy agents gemcitabine, cisplatin, and doxorubicin as well as targeted therapeutics such as topotecan, a topoisomerase inhibitor, and bevacizumab, a monoclonal antibody directed against vascular endothelial growth factor [7], [8]. While cisplatin is the most active and effective drug for ovarian cancer, resistance quickly develops, and many patients die with platinum-resistant cancer [9]. For placental cancer, methotrexate, a dihydrofolate reductase inhibitor, or Actinomycin D, a transcription inhibitor, is often used [10]. Combination therapy is common with a platinum-based compound given along with paclitaxel, a tubulin inhibitor [11], [12]. In addition to the compounds above, vaccine, antibody, and cell-based immunotherapies are being considered as treatments for gynecologic and other solid tumor cancers [13]. Despite great progress in developing novel solutions to improve the therapeutic outcome for treatment of gynecologic cancers, more work needs to be done to understand the varied responses to different drugs in patients with different gynecologic cancers [14].

To understand the diversity in compound efficacy across gynecologic cancers within individual cancer groups and identify new active compounds, we have screened 7914 compounds consisting of approved drugs and bioactive compounds using a quantitative high-throughput screening (qHTS) method against 11 unique gynecologic cancer cell lines derived from ovarian, cervical, and placental cancers. The results were analyzed to profile the chemotherapeutic activities of compounds against these gynecologic cancer cell lines. Our data demonstrate the commonality and diversity in responses of gynecologic cancers to the anticancer agents. We have also identified a group of non-anticancer compounds with antigynecologic cancer activities that can be further studied for target identification and drug development.

## Results

### Assay Development

**Figure 1:**
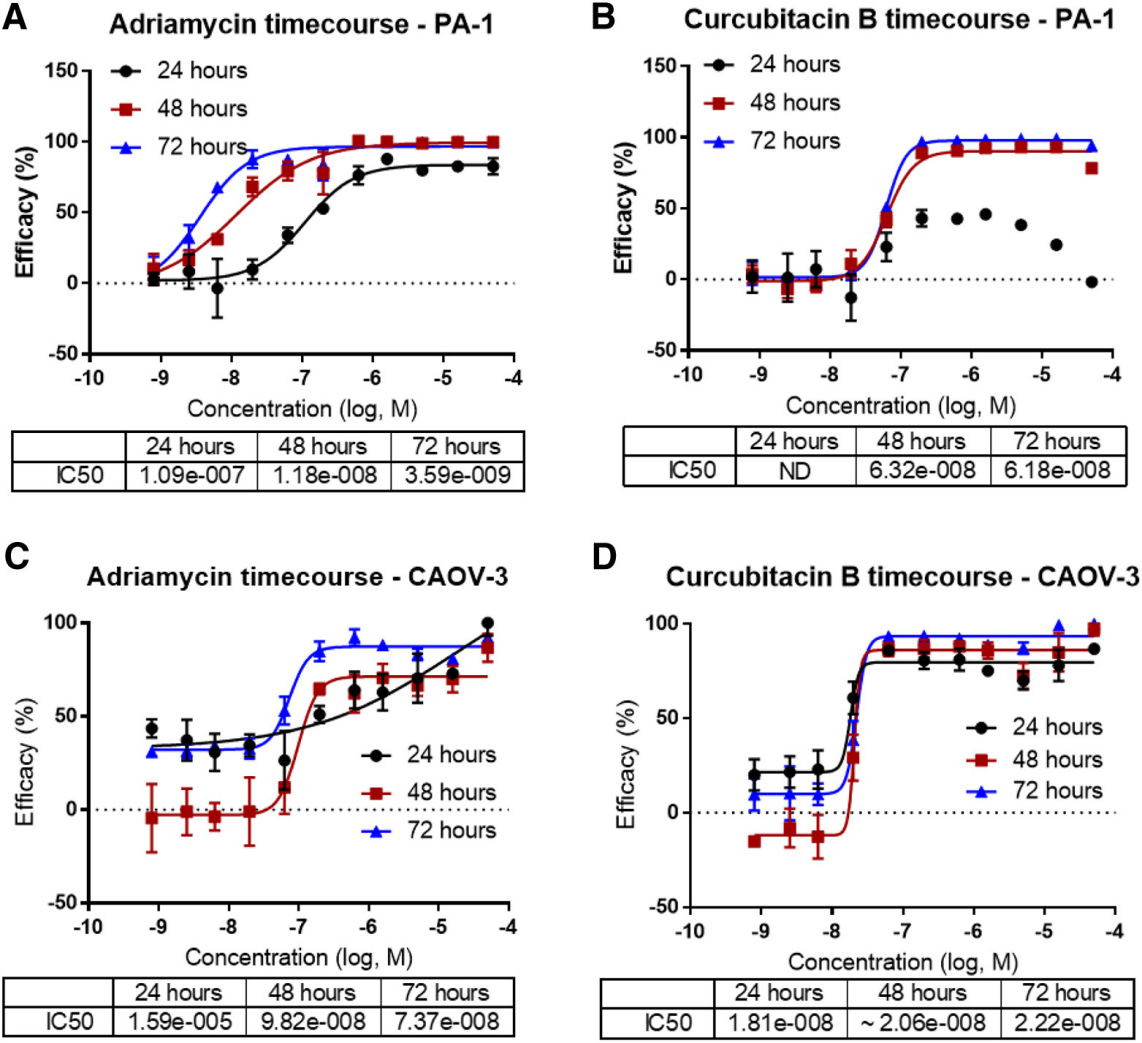
Assay development for qHTS screening of chemotherapeutic compounds. (A) Adriamycin time course dose-response curves for PA-1 cells from A, B, and C with IC_50_ determinations in the inset. (B) Curcubitacin B time course dose-response curves for PA-1 cells from A, B, and C with IC_50_ determinations in the inset. (C) Doxorubicin time course dose-response curve for CAOV-3 cells from A, B, and C with IC_50_ determinations in the inset. (D) Curcubitacin B time course dose-response curves for CAOV-3 cells from A, B, and C with IC_50_ determinations in the inset. Data points representing normalized mean ± S.D. (*n* = 4 wells per data point). Data were normalized to DMSO control (100% cell viability and lowest luminescence value among the 6 compounds (0% cell viability). Curves represent nonlinear regression curve fit with variable slope.

To determine the inhibitory effects of approved drugs and bioactive compounds on the common gynecologic cancer cell lines, 11 cell lines including 7 ovarian cancer lines (CAOV-3, SK-OV-3, SW 626, ES-2, PA-1, TOV-21G), 3 cervical cancer lines (HeLa, Ca Ski, and C-33 A), and 2 placental cancer lines (JAR, JEG-3) were used in the drug repurposing screen with HEK 293T cells as a control line to determine selectivity index of anticancer compounds (SI) [15], [16], [17] (Table 1; Supplementary Figure 1). The optimal assay conditions for the ATP content cell viability assay were determined in the ovarian PA-1 (Figure 1*A*, *B* and Supplementary Figure 2*A*-*C*) and CAOV-3 (Figure 1*C*, *D* and Supplementary Figure 2*D*-*F*) cell lines. Based on the assay optimization results, we used 1000 cells per well plated in 1536-well plates and a 48-hour incubation with compounds. The control compound activities (IC_50_) of adriamycin and curcubitacin B reached the steady state at this assay condition. Other standard-of-care (SOC) anticancer drugs examined during optimization included paclitaxel and topotecan [18] (Supplementary Figure 3*A*-*F*). Adriamycin and curcubitacin B were designated as the positive control compounds in the subsequent screens (Supplementary Figure 3*G*, *H*).

**Table 1:** Cell Lines Used in the OBGYN Cancer Chemotherapeutic Profiling

Cell Line	ATCC Catalog Number	Tissue Origin	Cancer Type or Cell Type	Mutations	Doubling time (+; days)
CAOV-3	HTB-75	Ovary	Adenocarcinoma	FAM123B, STK11, TP53	++
SK-OV-3	HTB-77	Ovary	Adenocarcinoma	CDKN2A, MLH1, PIK3CA, TP53	++
SW 626†	HTB-78	Ovary	Grade III, adenocarcinoma	APC, KRAS, TP53	++
ES-2	CRL-1978	Ovary	Clear cell carcinoma	B-RAF	++
PA1	CRL-1572	Ovary	Teratocarcinoma	NRAS	+
TOV-21G	CRL-11730	Ovary	Grade 3, stage III, primary malignant adenocarcinoma; clear cell carcinoma	TP53	+
TOV-112D*	CRL-11731	Ovary	Grade 3, STAGE IIIC, primary malignant papillary serous adenocarcinoma; endometrioid carcinoma	CTNNB1	++
OV-90*	CRL-11732	Ovary	Grade 3, stage IIIC, malignant papillary serous adenocarcinoma;	BRAF	+++
HeLa	CCL-2	Cervix	Adenocarcinoma	STK11, CTNNB1	+
Ca Ski	CRL-1550	Cervix	Epidermoid Carcinoma	STAG2	+++
C-33 A	HTB-31	Cervix	Carcinoma	RB1, PTEN, TP53	+
JAR†	HTB-144	Placenta	Choriocarcinoma	NA	+++
JEG-3	HTB-36	Placenta	Choriocarcinoma	NA	+++
HEK 293 T	CRL-3216	Embryonic kidney	Epithelial, noncancerous	NA	++

* These cell lines were used only in the primary screen.

† These cell lines were added for the confirmation screen.

### High-Throughput Compound Screening and Hit Confirmation

Following optimization, we next screened a collection of 7914 compounds including the FDA-approved drugs and bioactive compounds in 11 cancer cell lines shown in Table 1 (Supplementary Figure 1; Pubchem AID 1345084). From the primary screen, 256 hits were identified with the criteria of IC_50_ less than 10 μM, efficacy greater than 50%, and three-fold greater selectivity over the HEK 293T cells. From the primary screen, the signal-to-basal ratio was 9.30, coefficient of variation was 13.2% and *Z*' factor was 0.69 in the PA-1 cell line. For the CAOV-3 cell line, the signal-to-basal ratio was 9.86, coefficient of variation was 11.3%, and *Z*' factor was 0.71.

Among the primary hits tested in a follow-up screen (Pubchem AID 1345085), 205 compounds were confirmed using criteria of IC_50_ less than 30 μM, efficacy greater than 70%, and five-fold greater selectivity over HEK cells. A group of hits that were toxic to both cancer cells and HEK 293T cells was designated as the pan-toxic compounds (Supplementary Figure 4 and Table 2). The pan-cytotoxic compounds included panobinostat [19], givinostat [20], irestatin 9389 [21], NVP-BGT226 [22], vorinostat [23], TG-46 [24], NVP-TAE684 [25], and ponantinib [26]. The concentration-response curves for panobinostat (IC_50_ = 0.355 ± 0.268 μM; SI = 0.92 ± 0.57) and givinostat (IC_50_ = 3.50 ± 3.88 μM; SI = 1.74 ± 1.25), two HDAC inhibitors, are used as examples to illustrate the toxicity (Supplementary Figure 4).

**Table 2:** Hits with HEK293T Toxicity >50%, IC_50_ <30 μM, and CCL Efficacy >70%.

Toxic Compounds							
**Compound Name**	**FDA Approved**	**Compound Class**	**Target**	**Average SI**	**Average IC_50_ (μM)**		
Panobinostat	Yes; 2015	Antineoplastic; hydroxamate	Pan-HDAC	0.92 ± 0.57	0.355 ± 0.268		
Givinostat	No; in clinical trials	Antineoplastic; hydroxymate	Class I and II HDAC	1.74 ± 1.25	3.50 ± 3.88		
Irestatin 9389	No	Antineoplastic; diazole	IRE1 endonuclease	0.51 ± 0.20	3.52 ± 3.12		
NVP-BGT226	No; in clinical trials	Antineoplastic; imidazole quinoline	PI3K/mTOR	0.20 ± 0.26	5.34 ± 6.56		
Vorinostat	Yes; 2006	Antineoplastic; hydroxymate	HDAC	3.72 ± 2.24	5.50 ± 4.17		
TG-46	No	Antineoplastic	JAK2	10.5 ± 22.1	9.59 ± 6.87		
NVP-TAE684	No	Antineoplastic	ALK	4.87 ± 7.61	15.7 ± 10.0		
Ponantinib	Yes; 2012	Antineoplastic; pyridazine	Bcr-Abl	3.56 ± 4.85	15.9 ± 9.07		
Confirmation of HEK 293T toxicity Using an Independent Screen [84]							
**Compound Name**	**IC_50_**	**Efficacy (%)**	**Curve Class**	**Independent Screen**	**IC_50_**	**Efficacy (%)**	**Curve Class**
Panobinostat	0.21	82.6	−1.17	Confirmed toxic	0.162	85.5	−1.1
Givinostat	2.91	65.6	−1.17	Confirmed toxic	1.11	112	−1.1
Irestatin 9389	1.34	102	−1.1	Not toxic			
NVP-BGT226	0.258	106	−1.1	Confirmed toxic	0.0145	115	−1.1
Vorinostat	11.3	64.7	−1.93	Confirmed toxic	4.09	80.2	−1.2
TG-46	19.4	75.6	−2.1	Confirmed toxic	8.44	89.7	−2.15
NVP-TAE684	23.4	91.8	−2.1	Confirmed toxic	3.65	126	−2.1
Ponantinib	19.9	92	−2.1	Confirmed toxic	0.811	92.6	−1.1

Table depicting compounds that are toxic (EFFIC2ACY >70%) to all cell lines including HEK293T. Table shows compound name, FDA approval status, compound class, target, average selectivity, and average IC_50_ (μM).

### Chemotherapeutic Diversity Among 11 Gynecologic Cancer Cell Lines

**Figure 2:**
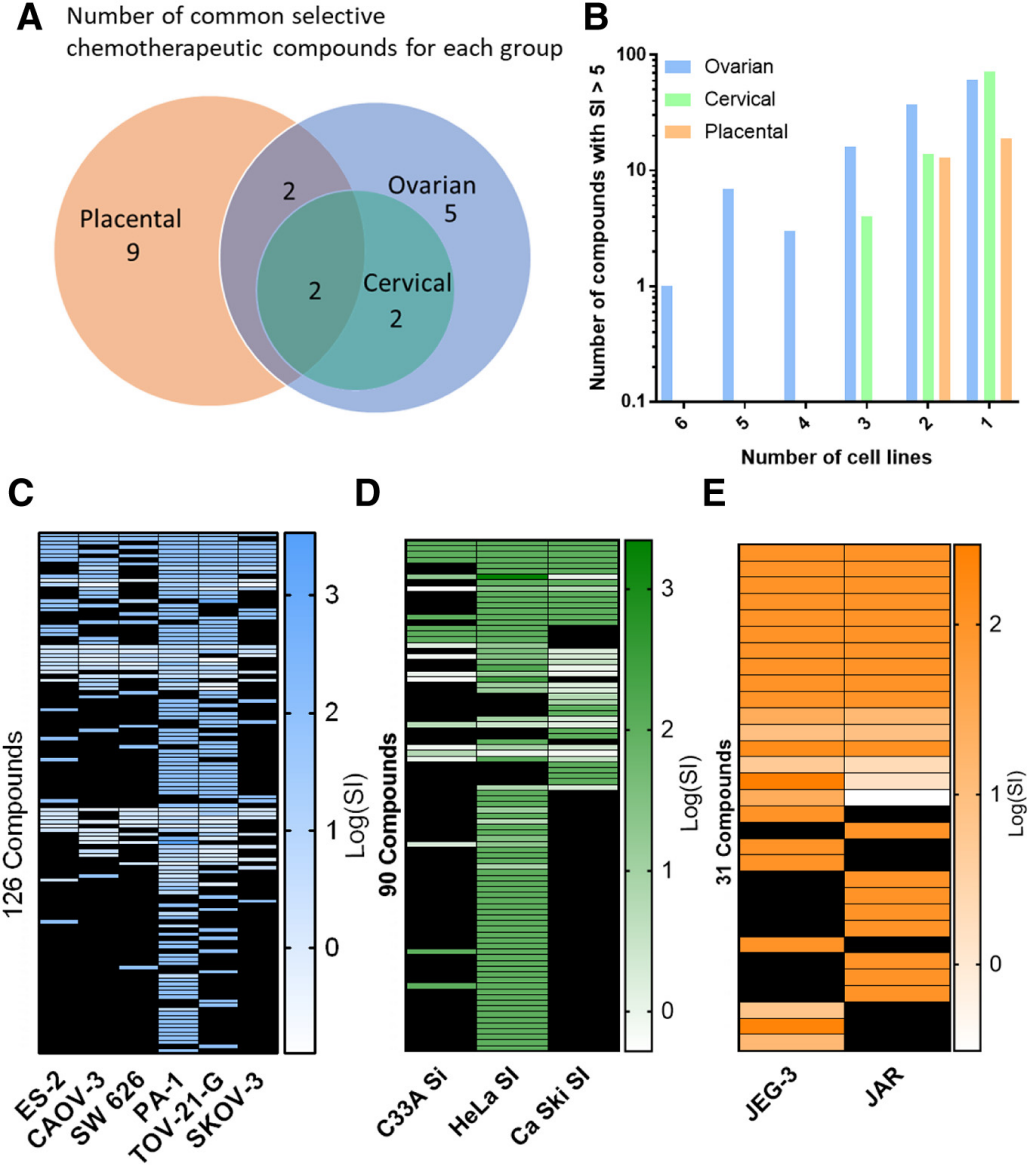
Chemotherapeutic diversity in cell line killing. (A) Venn diagram illustrating the number of selective compounds (efficacy >70%, IC_50_ <30 μM, SI >5) in each cancer group. Overlapping circles and number inset indicate number of compounds which are shared between the groups. Compound must be active in at least four of the six ovarian cancer cell lines to be considered ovarian cancer cell line selective. (B) Log scale bar graph depicting the number of compounds which had an SI >5 for each cancer line panel. Heat maps depicting the Log (SI) value for compounds active in at least one cell line with selectivity greater than five-fold for ovarian (C), cervical (D), and placental (E) cancer panels. Black boxes indicate no selectivity could be determined for that cell line.

**Figure 3:**
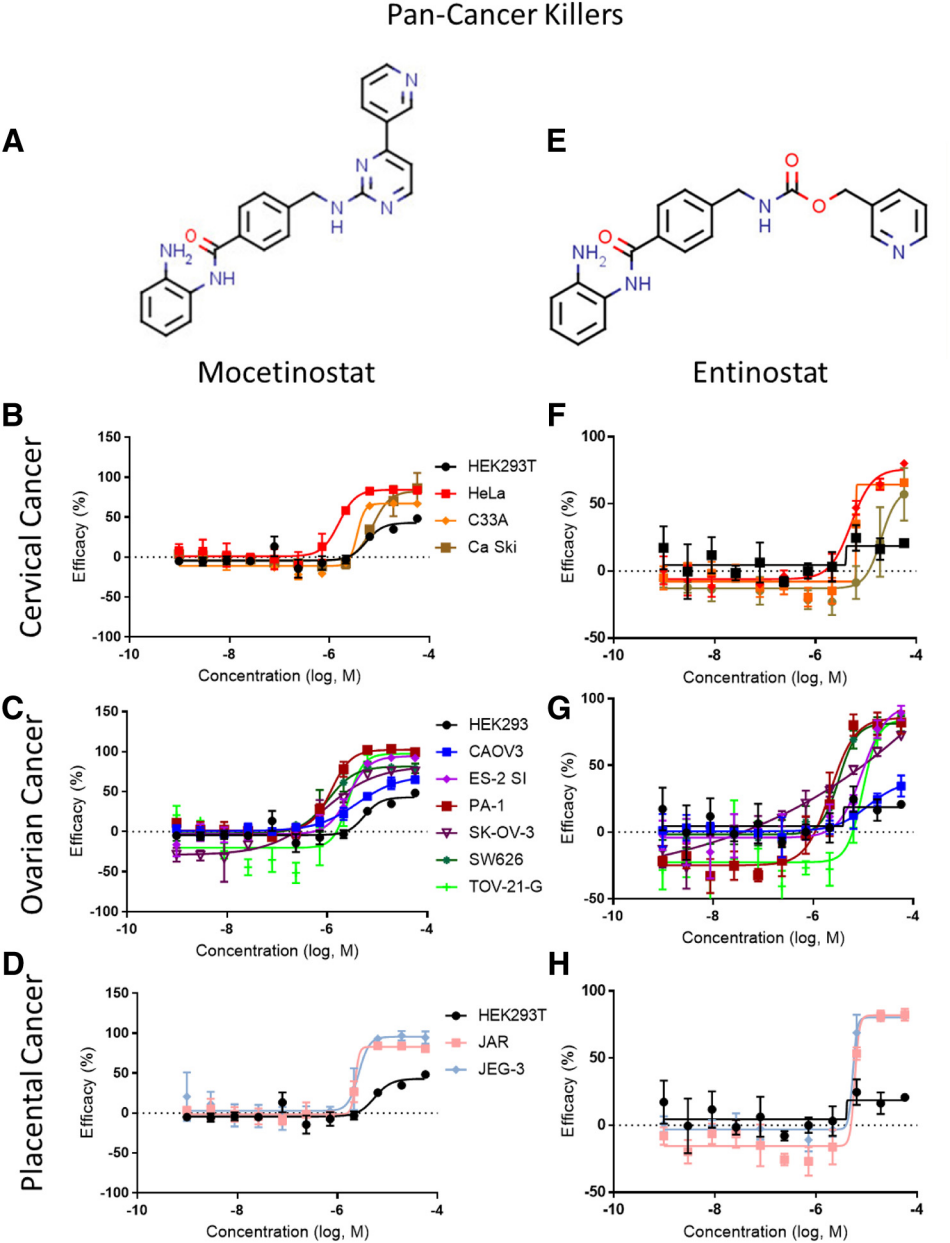
Pan-cancer killers. Chemical structures and dose-response curves for (A) mocetinostat and (E) entinostat, respectively, for (B, F) cervical, (C, G) ovarian, and (D, H) placental cancer cell lines. See Table 4 for the full list of the best compounds from the confirmation screen.

To further evaluate the 205 confirmed compounds in the 11 gynecologic cancer cell lines, we focused on the tissue types of these cancer cell lines to analyze the selectivity and diversity of compound activity. This analysis revealed two compounds, mocetinostat [27], [28], [29] (IC_50_ = 2.76 ± 1.98 μM; SI >100) and entinostat [30], [31] (IC_50_ = 7.11 ± 6.62 μM; SI >100), both class I HDAC inhibitors and in clinical trials, as pan-killers of all three cancer cell groups (Figures 2*A*, 3, and Table 3). The ovarian and placental cancer cell line selective inhibitors included actinomycin D [32] (IC_50_ = 0.78 ± 0.222 μM; SI >100), a DNA intercalator and common drug for GTD, and fedratinib [33] (IC_50_ = 13.1 ± 7.51 μM; SI >100), a JAK2 inhibitor (Supplementary Figure 6 and Table 3). The ovarian and cervical cancer cell line selective inhibitors included TG-89 [24] (IC_50_ = 11.2 ± 7.28 μM; SI >100), a JAK2 inhibitor, and CCT137690 [34] (IC_50_ = 20.0 ± 7.02 μM; SI >100), an Aurora kinase inhibitor (Supplementary Figure 7 and Table 3). For the individual cancer types, the top ovarian cancer cell selective inhibitor was fostamatinib [35] (IC_50_ = 6.24 ± 4.06 μM; SI >100), a Syk kinase inhibitor (Supplementary Figure 8*A*-*D* and Table 3). The top placental cancer line inhibitor was berberine [36], [37] (IC_50_ = 4.41 ± 0.662 μM; SI >100), an anti-parasitic alkaloid targeting Complex I of the mitochondrial respiratory chain and AP-1 machinery (Supplementary Figure 8*E*-*H* and Table 3). The cervical cancer selective inhibitory compounds found in our study were also active for the ovarian cancer cells.

**Table 3:** Diversity List of the Most Effective Compounds with IC_50_ <30 μM and CCL Efficacy >70%

Compound Name	FDA Approved	Compound Class	Target	Average SI	Average IC_50_ (μM)
Pan-GYN Cancer Cell Line Killer					
Mocetinostat	No; in clinical trials	Antineoplastic; 2-aminobenzamide	Class 1 HDAC	>100	2.76 ± 1.98
Entinostat	No; in clinical trial	Antineoplastic; 2-aminobenzamide	Class 1 HDAC	>100	7.11 ± 6.62
Ovarian + Placental Cancer Cell Line Killer					
Actinomycin D	Yes; 1964	Antibiotic; antineoplastic; multiple cancers	DNA intercalater	>100	0.78 ± 0.222
Fedratinib	No; in clinical trials	Antineoplastic	JAK2	>100	13.1 ± 7.51
Ovarian + Cervical Cancer Cell Line Killer					
TG-89	No	Antineoplastic	JAK2	>100	11.2 ± 7.28
CCT137690	No	Antineoplastic	Aurora kinase	>100	20.0 ± 7.02
Ovarian Cell Line Killer					
Fostamitinib	No; in clinical trials	Prodrug; Antineoplastic	Syk	>100	6.24 ± 4.06
AZ-960	No	NA	JAK2	>100	12.0 ± 7.75
WZ3146	No	NA	EGFR	>100	12.3 ± 8.52
AMG-Tie2-1	No	RTK inhibitor	Tie2	>100	15.9 ± 9.71
TAE226	No	NA	FAK	8.76 ± 2.40	5.32 ± 1.42
Placental Cancer Cell Line Killer					
Berberine	No	Antiparasitic/antifungal; benzylisoquinoline alkaloids	Complex I of mitochondrial respiratory chain	>100	4.41 ± 0.662
Nebupent	Yes; 1989	Antifungal	Topoisomerase II	>100	4.90 ± 1.02
PF-3845	No	NA	Fatty acid amide hydrolase	>100	9.31 ± 1.15
Cyclosporin A	Yes; 2000	Cyclic undecapeptide; immunosuppressant	Calcineurin	>100	16.7 ± 5.85
i-Bet-151	No	Pyrimidoindole	BET Bromodomain	>100	19.3 ± 9.13
WEHI-539	No	Benzothiazole-hydrazone	BCL-X(L)	>100	19.3 ± 5.11
Volasertib	No; in clinical trials	Dihydropteridinone	Plk1	131 ± 13.5	0.0709 ± 0.00735
Rotenone	No	Rotenoid	Complex I of mitochondrial respiratory chain	19.5 ± 6.26	0.0418 ± 0.0134
GSK461364	No; in clinical trials	Benzene sulfonamide thiazole	Plk1	8.81 ± 0.333	3.52 ± 0.133

Table shows compound name, FDA approval status, compound class, target, average selectivity, and average IC_50_ (μM). IC_50_ values are the mean of all cell lines that fulfill all criteria in the cancer grouping. Selectivity >100 indicates drug was “inactive” in HEK293T cells with efficacy <50%. No compounds were solely selective in cervical cancer.

Given that we included different numbers of cell lines for each of three gynecologic cancer groups, we assessed the number of compounds whose SI was greater than five (Figure 2*B*) in each group. Interestingly, while there were only 2 placental lines included in the study, 13 compounds reached an SI of 5 or greater in this group. Four compounds killed all three cervical cancer lines selectively, and only one compound, fedratinib, selectivity killed all six ovarian cancer lines. Fedratinib, one of the ovarian and cervical CCL selective inhibitors, has completed two Phase I clinical trials for solid tumors (ClinicalTrials.gov Identifier: NCT01836705; NCT01585623) but is not an FDA-approved drug. The heat maps for each cancer tissue group provided a high-level view of the SI for each compound that fulfilled the criteria (Figure 2*C*-*E*). These maps reveal that PA-1, TOV-21-G, and HeLa cells, the faster growing lines (Table 1), were more sensitive for qHTS as the compounds exhibited higher inhibitory activities.

### Single Cancer Cell Line Selective Compounds

**Figure 4:**
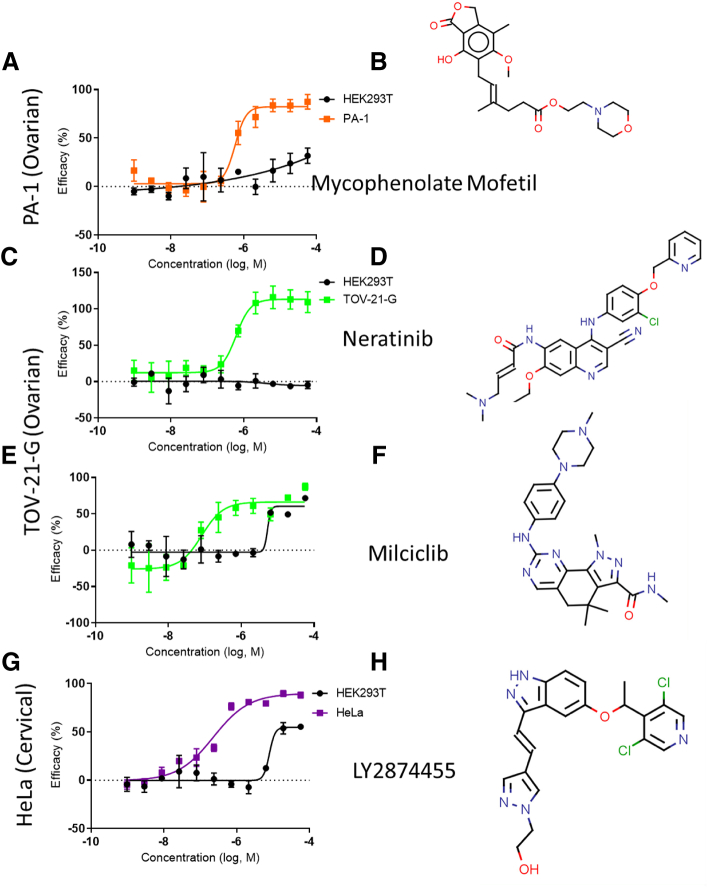
Representative compounds with selective toxicity and nanomolar potency in a single cell line. Chemical structure and dose-response curves for (A, B) mycophenolate mofetil in PA-1 cells, (C, D) neritinib in TOV-21-G cells, (E, F) milciclib in TOV-21-G cells, and (G, H) LY2974455 in HeLa cells. See Table 4 for the full list of the most effective compounds for a single cell line.

In addition to finding compounds with general antineoplastic activity, the selective inhibitory activities of compounds to individual cell lines were evaluated. We identified five compounds with selective inhibitory activities for PA-1, two compounds for TOV-21-G, and four compounds for HeLa (Figure 4 and Table 4). As mentioned above, these cell lines were the most susceptible to anticancer compounds because of their fast cell growth rates. We did not find selective compounds that only exhibited inhibitory activities to any of the eight remaining cancer cell lines individually. Since we performed a detailed analysis of the compounds' concentration-response curves, it helps to illustrate the significant differences in efficacy and potency between these lines and the control HEK 293T line. For PA-1, mycophenolate mofetil [38], an antifungal, was the most potent PA-1 suppressor (IC_50_ = 0.631 μM; SI >100). Neratinib [39] (IC_50_ = 0.619 μM; SI >100), an FDA-approved epidermal growth factor receptor (EGFR) inhibitor, and milciclib [40] (IC_50_ = 0.0897 μM (SI = 50.1), a CDK inhibitor, were the two most potent TOV-21-G inhibitors. The top HeLa suppressor was LY2874455 [41] (IC_50_ = 0.240; SI = 38.8) μM, a pan-FGFR inhibitor.

**Table 4:** Single Cell Line Selective Compounds with Nanomolar Potency

Compound Name	FDA Approved	Compound Class	Target	Avg SI	Avg IC_50_ (μM)
PA-1					
Mycophenolate mofetil	Yes; 2008	Immunosuppressant; prodrug	Inosine monophosphate dehydrogenase	>100	0.631
Pirarubicin	No; in clinical trials	Antineoplastic; anthracycline	DNA intercalater	14.6	0.839
Gimatecan	No; in clinical trials	Antineoplastic; quinolone akaloid	Topoisomerase I	12.5	0.0337
PHA-793887	No; in clinical trials	Antineoplastic	CDK2/1/4/9; GSK3β	12.3	0.194
Doxorubicin	Yes; 1993	Antineoplastic; anthracycline	DNA intercalater	7.02	0.576
TOV-21-G					
Neratinib	Yes; 2017	Antineoplastic	EGFR/Her2/Her4; P-glycoprotein	>100	0.619
Milciclib	No; in clinical trials	Antineoplastic	CDK; tropomyosin receptor kinase	50.1	0.0897
HeLa					
LY2874455	No; in clinical trials	Antineoplastic	Pan-FGFR	38.8	0.240
AZD3463	No	Antineoplastic	ALK/IGFR	30.3	0.638
NVP-TAE684	No	Antineoplastic	ALK	28.0	0.835
TAK 901	No; completed clinical trials	Antineoplastic	Aurora Kinase	12.6	0.699

Table shows compound name, FDA approval status, compound class, target, average selectivity, and average IC_50_ (μM). IC_50_ values are the mean of the cell line shown. Selectivity >100 indicates drug was “inactive” in HEK293T cells with efficacy <50%.

### Top Clinically Relevant Compounds

The results from our qHTS gynecologic cancer profiling revealed a diverse set of compounds with potencies ranging from the nanomolar to micromolar and different selectivity among three types of cancer tissues. We wanted to highlight these nanomolar compounds which may be useful to researchers and clinicians alike as these are the ones with anticancer activity to likely be far below their blood plasma concentrations, Cmax, in patients. We analyzed our data to uncover the number of compounds with less than 1 μM potency and greater than 70% efficacy regardless of selectivity. The data correspond with the similar trend for cytotoxic susceptibility in PA-1 (43 compounds), TOV-21-G (19 compounds), and HeLa (33 compounds) cells (Supplementary Figure 9*A*). We arranged the data to reflect how many cell lines have a number of compounds with a potency less than 1 μM. These data show that only one compound, the multitargeted HDAC inhibitor panobinostat (IC_50_ = 0.355 ± 0.268 μM; SI = 0.92 ± 0.57), exhibited sub-μM potency in every cancer cell line among 11 cancer cell lines tested (Supplementary Figure 9*B*).

To provide useful information with clinical relevance, we have analyzed the IC_90_s, the concentration needed to inhibit 90% of growth, of these potent compounds and correlated it to the relevant human plasma concentration of the drug. The most potent and effective drug we identified without taking selectivity into account was panobinostat. In one clinical trial, panobinostat's median Cmax human plasma concentration after oral administration was measured to be 0.061 μM (range 0.038-0.119 μM) [42]. In an independent study, intravenous administration of panobinostat at doses from 1.20 to 20.0 mg/m^2^ resulted in a Cmax of 0.107 to 2.24 μM [43]. The IC_50_ of panobinostat for the ovarian, cervical, and placental lines in our study is 0.343, 0.224, and 0.516 μM, respectively. The IC_90_ average for all cell lines is 0.719 μM, within the range of the intravenous, but not oral, Cmax values.

Bortezomib, a 20S proteasome inhibitor, exhibited an average IC_50_ of 0.150 μM with good efficacy in 8 of the 11 cancer cell lines excluding SKOV-3, HeLa, and JAR. Its average IC_90_ was 0.218 μM, well within the intravenous dose Cmax of 580 nM [44]. Elesclomol, a ROS inducer, was active in six cell lines with an IC_50_ of 0.173 μM and an IC_90_ of 0.283 μM. The Cmax of elesclomol in a clinical trial ranged from 1.32 to 12.84 μM with doses of 44 to 438 mg/m^2^ [45]. Thus, elesclomol is a good clinically relevant candidate for gynecologic cancers. Actinomycin D, mentioned previously as an FDA-approved drug for multiple cancers, exhibited nanomolar potency against six cell lines as well while maintaining high selectivity for cancer cell lines. The average IC_90_ for Actinomycin D in our study against ES-2, CAOV3, PA-1, TOV-21-G, SK-OV-3, and Ca Ski was 512 nM, while the Cmax in a pediatric population can range from 4 to 97.2 nM after 15 minutes of exposure to the drug [46]. Another trial measured a Cmax ranging from 2.5 to 79 nM, indicating that the IC_90_ identified in our study is several-fold above what can be achieved in human blood plasma [47]. The extended comparison of IC_90_ to Cmax values for the most promising clinical candidates from Supplementary Figure 9 is presented in Table 5.

**Table 5:** IC_90_ and Cmax Values for Nanomolar Potent Compounds

Compound Name	FDA Approval	IC_90_ (μM)	Cmax (μM)	Cell Lines Active	Reference
Panobinostat (LBH589)	Yes; 2015	0.719	0.107-2.24	11	[42], [43]
Bortezomib	Yes; 2003	0.218	0.580	8	[44]
Elesclomol (STA-4783)	No; in clinical trials	0.283	1.32-12.84	6	[45]
CEP-18770 (Delanzomib)	No; in clinical trials	0.391	0.214-1.35	6	[85]
BI-2536	No; in clinical trials	0.0397	1.61	4	[86]
SN-38	No; in clinical trials	0.592	0.086	4	[87]
Gedatolisib	No; in clinical trials	6.80	16.2	4	[88]
Gimatecan	No; clinical trials completed	0.275 ± 0.028	0.103 -0.349	4	[89]
Volasertib	No; in clinical trials	0.090	1.60-2.26	4	[90]

Table shows compound name, FDA approval status, average IC_90_ (μM), Cmax, and the number of cell lines for which each compound is active.

## Discussion

Heterogeneous responses in gynecologic cancers to chemotherapeutic drugs make it challenging to predict the drug's clinical effectiveness. This heterogeneity arises from differences in patient genetic background, patient age, tumor microenvironment, treatment regimen, and intrinsic resistance to drug therapy. In general, overall cancer incidence and death rates for women have been falling since the 1930s [2], [48]. Ovarian cancer death rates peaked in 1970 at 10.6 deaths per 100,000 women and in 2015 stood at 7.1 deaths per 100,000 women [48]. Uterine cancer, including cervix and corpus, however, killed 37.6 women per 100,000 in 1932 and now stands at 7.1 deaths per 100,000 women [48]. The last few years have seen a slight rise in death rates for uterine cancers from 6.5 in 2009 to 7.1 in 2015 [48]. Ovarian cancer’s 5-year survival rates remain among the lowest survival rates of all female cancer types, rising slowly from 1975 (36% survival) to 2013 (47% survival) [49]. Furthermore, the development of selective chemotherapeutics that are selectively toxic to cancer cells is an ongoing mission in the cancer therapeutic research field. Understanding the differences and similarities in the chemotherapeutic responses of different gynecologic cancer cell types through chemotherapeutic profiling can aid in the development of safer, more effective therapies for these types of cancers. In this work, we have utilized a qHTS approach to profile the chemotherapeutic responses and selectivity of 11 gynecologic cancer cell lines to known chemotherapeutic molecules as well as other approved drugs and biologically active compounds.

We assessed the cytotoxicity of 7914 compounds consisting of approved drugs, drug candidates tested in clinical trials, and bioactive compounds in six ovarian, three cervical, and two placental cancer cell lines. Two Class I HDACIs, mocetinostat and entinostat, were identified and confirmed as pan-gynecologic cancer inhibitors with high degrees of efficacy and selectivity (SI >100) in all three cancer groups. Interestingly, we did not find other HDACIs to be as selective except for these two. Indeed, panobinostat, givinostat, and vorinostat, three other HDAC inhibitors, were found to be equally toxic to HEK 293T cells in our screens in addition to suppressing the 11 gynecologic cancer cell lines. HDACIs prevent the removal of acetyl groups on histone lysines and, in effect, open chromatin structure to modulate gene expression [50]. Generally, epigenetic pathways are modified by HDACIs to cause changes in the expression of genes which can induce cell-cycle arrest or apoptosis [51]. In addition to regulating histone acetylation, HDACIs can inhibit the function of nonhistone effectors such as transcription factors to modulate gene expression.

In order to advance the compounds identified from a drug repurposing screen to potential clinical trials, the blood plasma concentration of the drug should be a few-fold higher than its IC_50_ value or similar to or below its IC_90_ value in the cells of the newly identified indication. We researched the human Cmax values of our most broadly potent compounds and compared them to the experimental IC_90_ values in this study. In most cases, our experimental IC_90_ is at or below the human plasma concentration, indicating that the effective drug concentration against the new indication is achievable in patients. Mocetinostat has a Cmax of approximately 21.4 μM at 10 mg/kg and 75.7 μM at 40 mg/kg in humans [52], while entinostat in humans reached a Cmax of 0.46 μM with 15 mg [53]. For mocetinostat, whose IC_50_ in our work was found to be 2.76 ± 1.98 μM, this indicates that the Cmax is well above its anticancer activity. For entinostat, however, although the patient Cmax is significantly lower than the average IC_50_ achieved in our study (7.11 ± 6.62 μM) for gynecologic cancers, its *in vivo* activity could possibly be achieved in higher doses or with compound structure-activity optimization. It is possible that the low toxicity of mocetinostat and entinostat is due to their specific HDAC isotype selectivity for certain HDACs. Both are class I HDAC inhibitors but exhibit varying IC_50_s for specific HDACs. For example, mocetinostat was found to inhibit only HDAC 1/2/3/11 at low micromolar potency or below [54]. On the other hand, entinostat exhibited submicromolar potency against HDAC 1/2/3 only [55]. Their similar isotype selectivity profiles correlate with their similar *in vitro* effects against gynecologic cancers in our study. This HDAC isotype selectivity may be related to the drugs' activity against the gynecologic cancer cell lines as HDAC 1/2/3 have been implicated in ovarian tumor malignancy and growth [56], while HDAC2 is overexpressed in cervical cancer carcinogenesis [57].

We also identified single cell line selective compounds with submicromolar potency and high selectivity for PA-1 (ovarian), TOV-21-G (ovarian), and HeLa (cervical), which could be due to their faster growth rates compared to other cancer cell lines and the cell cycle–interrupting nature of many compounds. Empirically, cells which cycle faster are more susceptible to interruptions of cell growth at different cycle stages [58]. However, certain drugs may act by disrupting specific cycle stage progression, *i.e.*, G_0_ to G_1_ [59]. It is known that certain drugs are specific to certain phases. For example, 5-fluorouracil interrupts S phase by reducing thymidylate content for DNA synthesis [60], docetaxel interrupts M phase by preventing microtubule polymerization [61], [62], and seliciclib interrupts G1 phase by inhibiting CDKs 2/7/9 [63]. In this screen, PHA-793887 [64], a CDK2/1/4/9 inhibitor, was found to be potently toxic to PA-1 specifically, while milciclib [65], another CDK2 selective inhibitor, was specifically toxic to TOV-21-G with nanomolar potency. Both of these two CDK inhibitors suppress the cell growth phase.

The control cell line in this study, HEK 293T, is a normal human cell line originating from human embryonic kidney cells that is typically used as control cell line. The selectivity values determined in this study were relevant to the cytotoxicity of the compounds in HEK 293T cells. Given a different control line, the resulting selectivity may be different. The *in vivo* toxicity of compounds may also be different from the *in vitro* SI data. The selectivity reported here is for reference, and it should be noted that it cannot replace the data obtained from *in vivo* drug safety experiments and in clinical trials. We acknowledge the unequal numbers of lines for each cancer group (ovarian, cervical, and placental). Having fewer lines in one group will potentially increase the number of compounds that are pan-killers for that particular group. This is evident in the larger number of compounds that killed both placental lines as compared to the number of compounds that killed all six ovarian lines.

The results of this study warrant further investigation into the different responses cancers have to similar classes of compounds. Here, different HDAC inhibitors exhibit differential selectivity. This could possibly be due to differences in HDAC class specificity, with some inhibitors targeting class I HDACs preferentially to class II HDACs, for example [66]. Of the 19 compounds found to be pan-killers for all or some of the cancer groups, only three are FDA-approved drugs including Actinomycin D, nebupent [67], and cyclosporin A [68]. Of these, only Actinomycin D is an FDA-approved antineoplastic, while nebupent is an antifungal targeting Topoisomerase II and cyclosporin A is an immunosuppressant targeting calcineurin. Actinomycin D has been used as an alternative chemotherapeutic regimen for ovarian cancer [69] and GTD (placental cancer) [12]. As nebupent disrupts mitotic activities, it has been researched as an antineoplastic agent *in vivo* against adenocarcinomic human alveolar basal epithelial (A549 cells) and colorectal carcinoma (HCT116 cells) xenografts in combination with chlorpromazine [70] but is not used as an anticancer therapy in the clinic nor has it been used in the study of gynecologic cancer. Lastly, cyclosporin A showed no efficacy for platinum-resistant ovarian cancer in one Phase II trial [71]. In another trial studying drug-resistant gynecologic cancer, however, patients had an overall response rate of 29% after cyclosporin A treatment, and it was well tolerated [72]. Future work will seek to understand chemotherapeutic selectivity in more advanced models such as tumor spheroids, organoids, and *in vivo* xenograft models that could provide more physiologically relevant data on tumor killing.

Drug resistance to chemotherapy is a common cause for relapse and recurrence of many different types of cancers [73], [74]. Platinum resistance is a common form of drug resistance in ovarian cancer with several suspected underlying causes including CDK expression, Akt signaling, and EGFR expression [75], [76], [77]. Our group recently published a set of compounds that were able to overcome cisplatin resistance in several platinum-resistant ovarian cancer cell lines when given alone and in combination with cisplatin [78]. The newly identified compounds in this study against gynecologic cancers can be used to further study the drugs' synergistic effects with the SOC anticancer drugs. Therefore, some of our hits may be of interest in studying how to overcome drug resistance in ovarian, cervical, and placental cancers using the synergistic drug combination with the SOC anticancer drugs.

In conclusion, the compounds identified and confirmed in this drug repurposing screen and profiling can be used to further investigate their utility in the treatment of gynecological cancer, especially for multidrug-resistant cancer patients. We demonstrate here the variability and heterogeneous responses of gynecologic cancer cells to anticancer drugs that may be related to patient genetic background, age, intrinsic drug resistance, and cancer aggressiveness. Two HDAC inhibitors identified in this study, mocetinostat and entinostat, may have high clinical relevance and can be moved to clinical trials as *bona fide* gynecologic cancer therapeutics. Indeed, entinostat in combination with avelumab is already in Phase I/II clinical trials for epithelial ovarian cancer, peritoneal cancer, and fallopian tube cancer (ClinicalTrials.gov: NCT02915523). Likewise, despite its toxicity to HEK 293T cells, panobinostat may be further studied in *in vivo* experiments due to its extremely high potency in gynecologic cancers. In conclusion, the chemotherapeutic profiling in individual cancer cells is an effective method to reveal the best anticancer therapeutics that might be particularly useful for those cancers with multidrug resistance, poor prognosis, and survival rates.

## Methods

### Reagents

DMEM (11965092), penicillin/streptomycin (15140163), and TrypLE (12605010) were purchased from Life Technologies. FBS (SH30071.03) was purchased from HyClone (SH30071.03). ATPlite (6016739) was purchased from Perkin Elmer.

### Cell Lines

The following cell lines were purchased from ATCC: CAOV-3 (ovarian adenocarcinoma; HTB-75), SK-OV-3 (ovarian adenocarcinoma; HTB-77), SW 626 (ovarian adenocarcinoma; HTB-78), ES-2 (ovarian clear cell carcinoma; CRL-1978), PA-1 (ovarian teratocarcinoma; CRL-1572), TOV-21G (ovarian clear cell carcinoma; CRL-11730), HeLa (cervical adenocarcinoma; CCL-2), Ca ski (cervical epidermoid carcinoma; CRL-1550), C-33 A (cervical carcinoma; HTB-31), JAR (placental choriocarcinoma; HTB-144), JEG-3 (placental choriocarcinoma; HTB-36), and HEK 293T (embryonic kidney fibroblast; CRL-3216).

### Cell Culture

Cells were kept in cryovials frozen at −150°C and thawed quickly in a 37°C water bath. A total of 1.5 million cells were seeded into T-225 flasks and subcultured once using TrypLE before freezing down for future experiments. For all assays, cells were seeded at 1000 cells per well into white, solid-bottom 1536-well plates using a Thermo Fisher Multidrop Combi reagent dispenser.

### ATP Content Assay for Cell Viability, Growth Rate, and Positive Control Determination

The ATPlite luminescence assay system assay kit was used to determine cell viability. The reagent was reconstituted and prepared as described by the manufacturer. To measure the cell death caused by the compounds, cells were cultured in 4 μl of media for 16 hours at 37°C with 5% CO_2_ in assay plates, followed by the addition of DMSO or 16 SOC chemotherapeutic compounds dissolved in DMSO. SOC compounds were dosed at 11 concentrations (1:3 dilution) in quadruplicate from 57.5 μM to 0.977 nM using the automated Wako 1536 Pin Tool workstation and incubated at 37°C with 5% CO_2_ for 24, 48, or 72 hours. Four microliters of ATPlite, the ATP monitoring reagent, was then added to each well of the assay plates using the Multidrop Combi reagent dispenser followed by incubation for 15 minutes at room temperature. The resulting luminescence was measured using the ViewLux plate reader. Data were normalized for each drug using the largest luminescence value as 100% full cell viability (0% cell killing) and to the smallest luminescence value 0% viability (100% cell killing).

### Large-Scale Compound Screening and Follow-Up

A qHTS [79], in which each compound was assayed in five concentrations (0.092, 0.46, 2.3, 11.5, and 57.5 μM), was performed for the primary compound screen using the NPC [80] and NPACT drug libraries at NCATS. The OBGYN cancer and HEK 293T control cells were seeded into 1536-well assay plates at 1000 cells per 4 μl/well and incubated at 37°C in 5% CO2 for 48 hours. The ATPlite assay to determine the IC_50_ values for each compound was conducted as described above. Plates were processed on the fully integrated Kalypsys robotic system. Hits were selected from the primary screen for follow-up confirmation, dosed in triplicate at 11 concentrations (1:3 dilution) from 57.5 μM to 0.977 nM, and incubated for 48 hours, and the ATPlite assay was used to determine the IC_50_ values.

### Statistical Analysis

Data analysis was performed using Microsoft Excel, and figures were generated using Prism Graphpad 7.0. In-house qHTS data normalization, correction, curve fitting, and classification were performed using custom programs developed at NCATS [81], [82], [83]. All data presented as mean ± S.D. unless otherwise stated.

## Data Availability Statement

Data have been submitted to Pubchem. Primary Screen AID: 1345084. Confirmatory Screen AID: 1345085.

## Appendix A Supplementary data

Supplementary Figure 1. Assay development for qHTS screening of chemotherapeutic compounds. ATPlite luminescence from PA-1 ovarian teratocarcinoma cells treated with 10 compounds for 24 (A), 48 (B), and 72 hours (C) in dose-response format including SOC drugs used in the clinic. ATPlite luminescence from CAOV-3 ovarian adenocarcinoma cells treated with 16 compounds for 24 (D), 48 (E), and 72 hours (F) in dose-response format including SOC drugs used in the clinic. Data points representing normalized mean ± S.D. (*n*=4 wells per data point). Data were normalized to DMSO control (100% cell viability and lowest luminescence value among the 6 compounds (0% cell viability). Curves represent non-linear regression curve fit with variable slope. Supplementary Figure 2. qHTS Assay Workflow for chemotherapeutic profiling of OBGYN cancer cell lines. Supplementary Figure 3. HEK 293T control assay development. ATPlite luminescence from HEK 293T embryonic kidney fibroblasts cells treated with 10 compounds for 24 (A), 48 (B), and 72 hours (C) in dose-response format including SOC drugs used in the clinic. (D) Doxorubicin time course dose-response cell viability curves for PA-1 cells from A, B, and C with IC_50_ determinations in the inset. (E) Curcubitacin B time course dose-response curves for PA-1 cells from A, B, and C with IC_50_ determinations in the inset. (F) Log (selectivity index) heat map calculated using the 48-hour time point IC_50_ values for HEK 293T cells divided by the IC_50_ values for PA1 and CAOV-3 cells. Table is organized according to the most selective to the least selective compounds. Black panels indicate that no IC_50_ could be determined from the linear regression curve fit. (G) Chemical structure of doxorubicin. (H) Chemical structure of curcubitacin B. Data points representing normalized mean ± S.D. (*n*=4 wells per data point). Data were normalized to DMSO control (100% cell viability and lowest luminescence value among the 16 compounds (0% cell viability). Curves represent nonlinear regression curve fit with variable slope. Supplementary Figure 3. Comparison of IC50 and efficacy values for eight toxic compounds. The eight compounds in this dataset were filtered with the following dose-response parameters: efficacy >70% and IC_50_ <30 μM in all of the cell lines in addition to having toxicity in HEK293T >50%. (A) Comparison of CCL (circles) and HEK293T (triangles) average IC_50_ (μM) vs. average efficacy (%) along with linear regression curves and the 95% CI. (B) Efficacy values of eight toxic compounds for each cancer cell line shown as box and whisker plots, with the box representing the upper 25% and lower 75%, the middle line representing the median, the “+” representing the mean, and the whiskers representing the 5 and 95 percentiles. Outliers above 95 and below 5 percentile range shown as circles. (C) IC_50_ (log, M) of eight toxic compounds for each cancer cell line shown as box and whisker plots, with the box representing the upper 25% and lower 75%, the middle line representing the median, the “+” representing the mean, and the whiskers representing the 5 and 95 percentiles. Outliers above 95 and below 5 percentile range shown as circles. (d) The IC_50_ (μM) (red circles) and efficacy (%) (black circles) shown for each compound sorted from the largest to smallest response. Supplementary Figure 4. Two representative compounds with toxicity in all cell lines. Chemical structures and dose-response curves for (A) panobinostat and (E) givinostat, respectively, for (B, F) cervical, (C, G) ovarian, and (D, H) placental cancer cell lines. See Table 2 for the full list of effective compounds toxic to all cell lines. Supplementary Figure 5. Confirmation of compound toxicity in HEK293T. (A) Comparison of IC_50_ (log, M) and (B) efficacy (%) values for compounds toxic to all cell lines between two independent screens. Primary and secondary assays used ATPlite. Confirmation assay used CellTiterGlo. Missing values in A were compounds not present in the CellTiter Glo confirmation screen drug library. Supplementary Figure 6. Ovarian and placental cancer killers. Chemical structures and dose-response curves for (A) actinomycin D and (E) TG-101348, respectively, for (B, F) cervical, (C, G) ovarian, and (D, H) placental cancer cell lines. See Table 4 for the full list of the best compounds from the confirmation screen. Supplementary Figure 7. Ovarian and cervical cancer killers. Chemical structures and dose-response curves for (A) TG-89 and (E) CCT137690, respectively, for (B, F) cervical, (C, G) ovarian, and (D, H) placental cancer cell lines. See Table 4 for the full list of the best compounds from the confirmation screen. Supplementary Figure 8. Cancer group selective cancer killers. Chemical structure and dose-response curves for representative ovarian cancer killer (A) fostamitinib and placental cancer killer (E) nebupent for (B, F) cervical, (C, G) ovarian, and (D, H) placental cancer cell lines. See Table 4 for the full list of the best compounds from the confirmation screen. Supplementary Figure 9. Subnanomolar compound diversity among different cancer types. (A) Bar graph depicting each cell line with the number of nanomolar compounds with greater than 70% efficacy. (B) Bar graph depicting the number of cell lines killed by compounds with <1 nM IC_50_. Image 1
