# Supplementary material for: Quantitative Chemotherapeutic Profiling of Gynecologic Cancer Cell Lines Using Approved Drugs and Bioactive Compounds
Source: Transl Oncol. 2018 Dec 18;12(3):441–52. doi: 10.1016/j.tranon.2018.11.016 (PMC6302136; doi:10.1016/j.tranon.2018.11.016)

## Slide 1
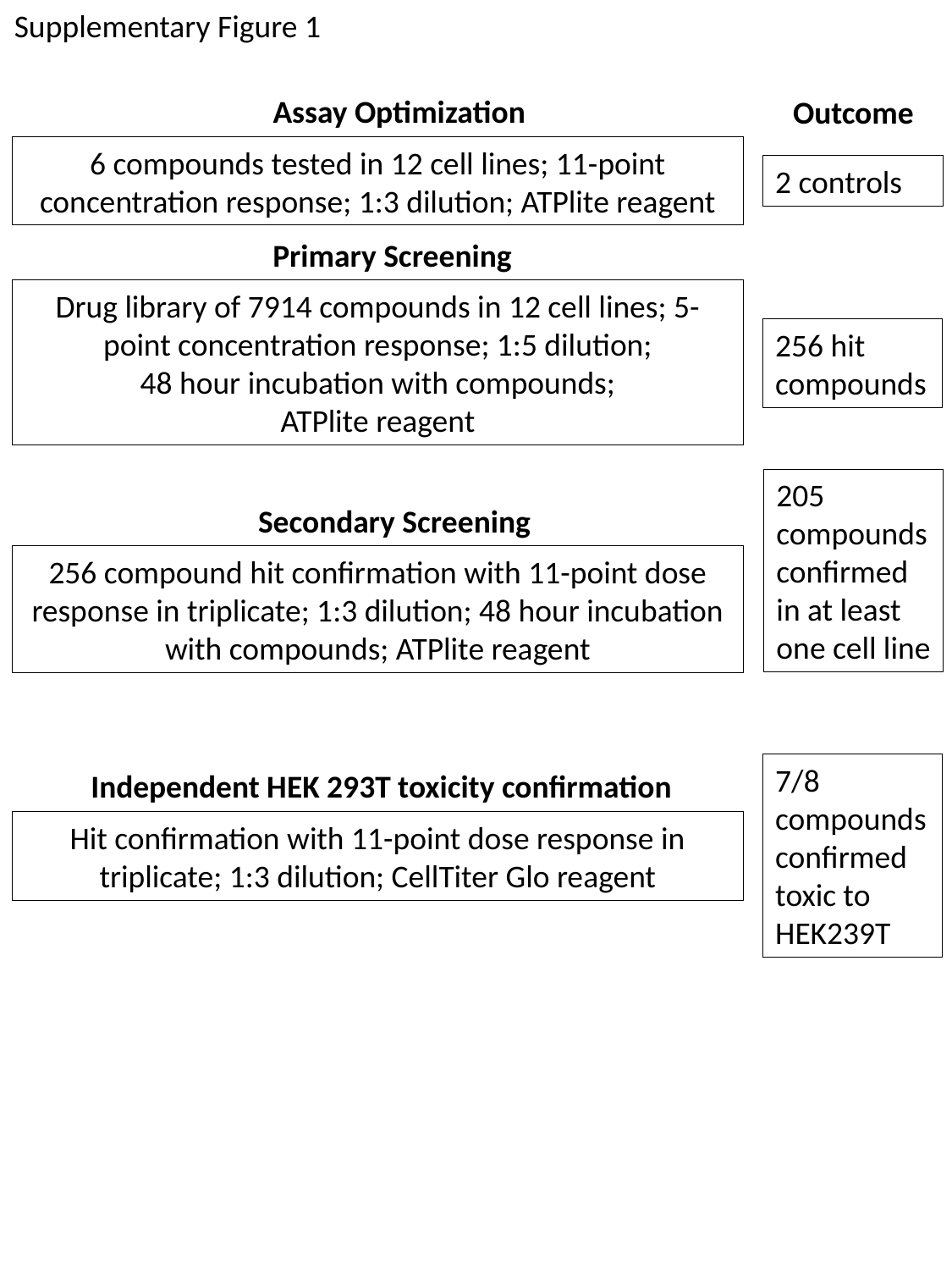

Supplementary Figure 1
Assay Optimization
Outcome
6 compounds tested in 12 cell lines; 11-point concentration response; 1:3 dilution; ATPlite reagent
2 controls
Primary Screening
Drug library of 7914 compounds in 12 cell lines; 5-point concentration response; 1:5 dilution;
48 hour incubation with compounds;
ATPlite reagent
256 hit compounds
205 compounds confirmed in at least one cell line
Secondary Screening
256 compound hit confirmation with 11-point dose response in triplicate; 1:3 dilution; 48 hour incubation with compounds; ATPlite reagent
7/8 compounds confirmed toxic to HEK239T
Independent HEK 293T toxicity confirmation
Hit confirmation with 11-point dose response in triplicate; 1:3 dilution; CellTiter Glo reagent

## Slide 2
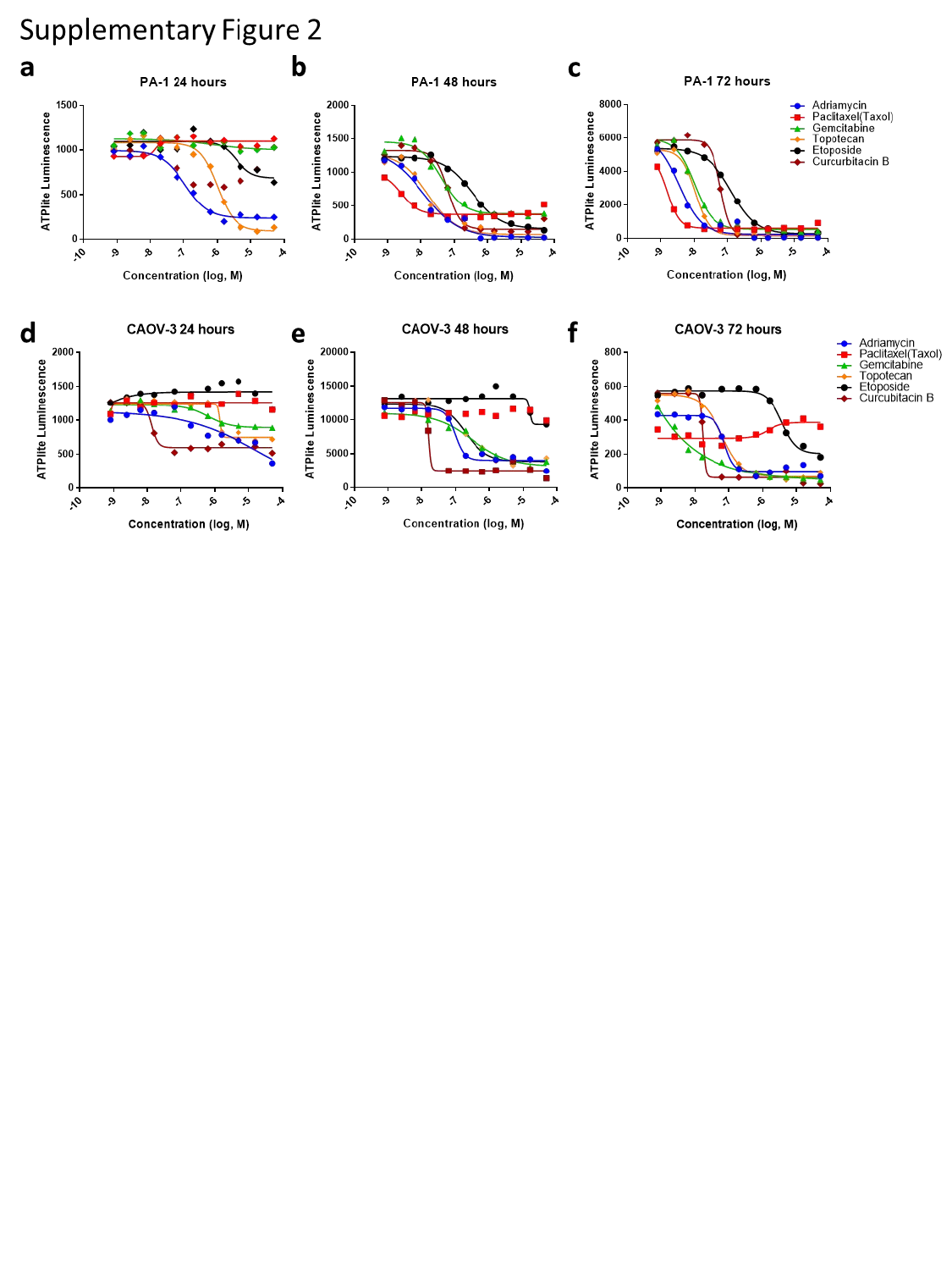

## Slide 3
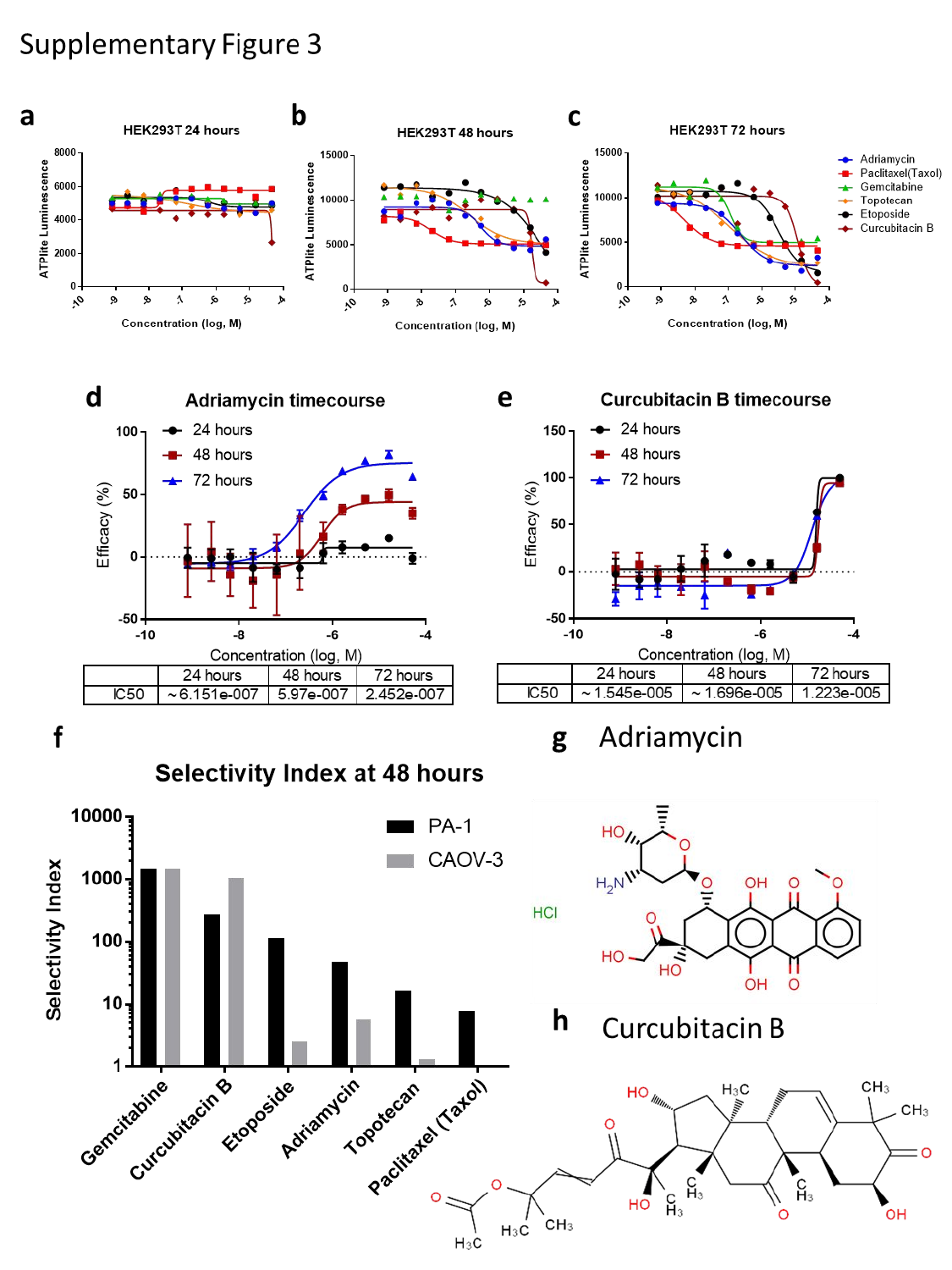

## Slide 4
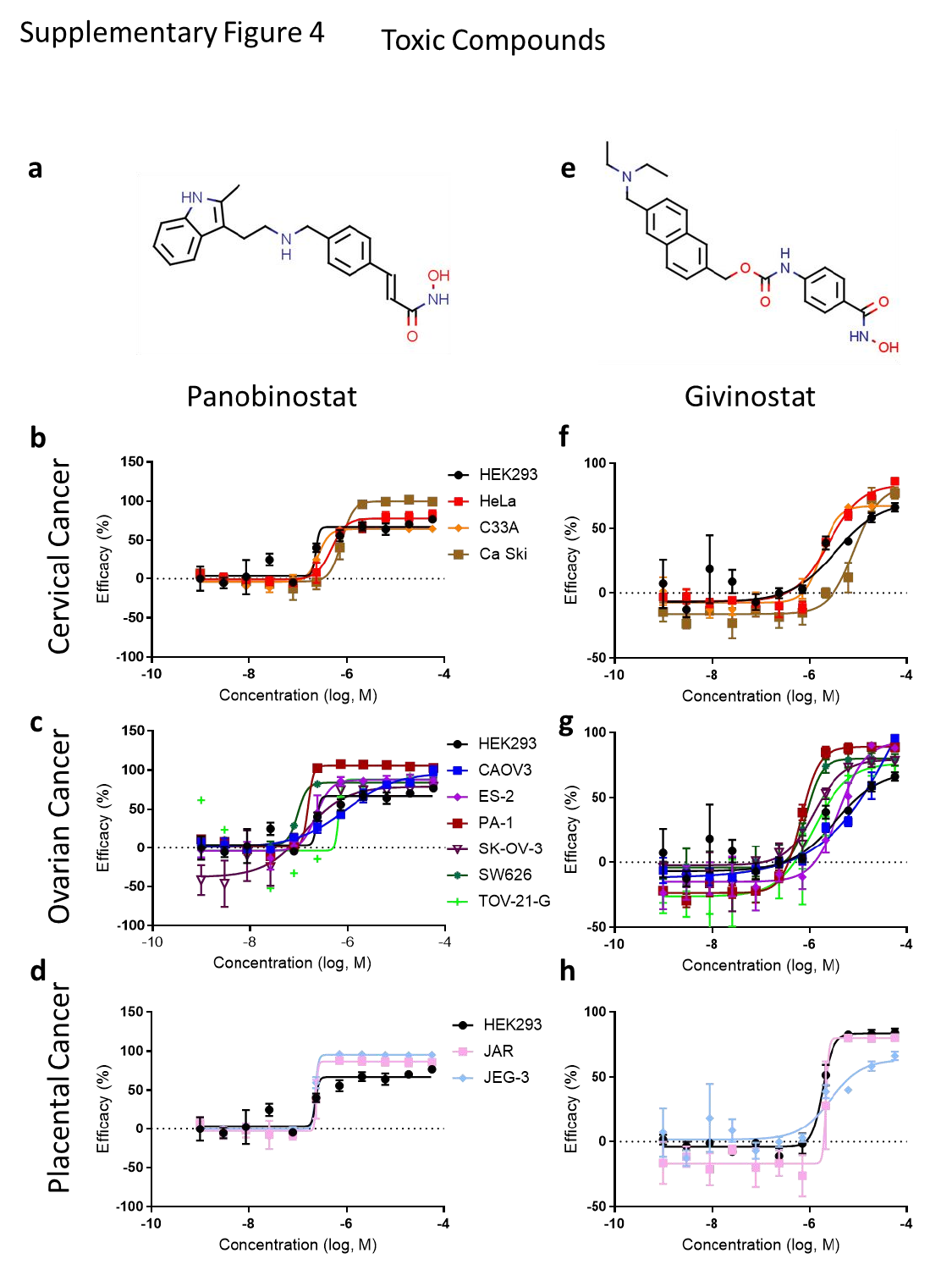

## Slide 5
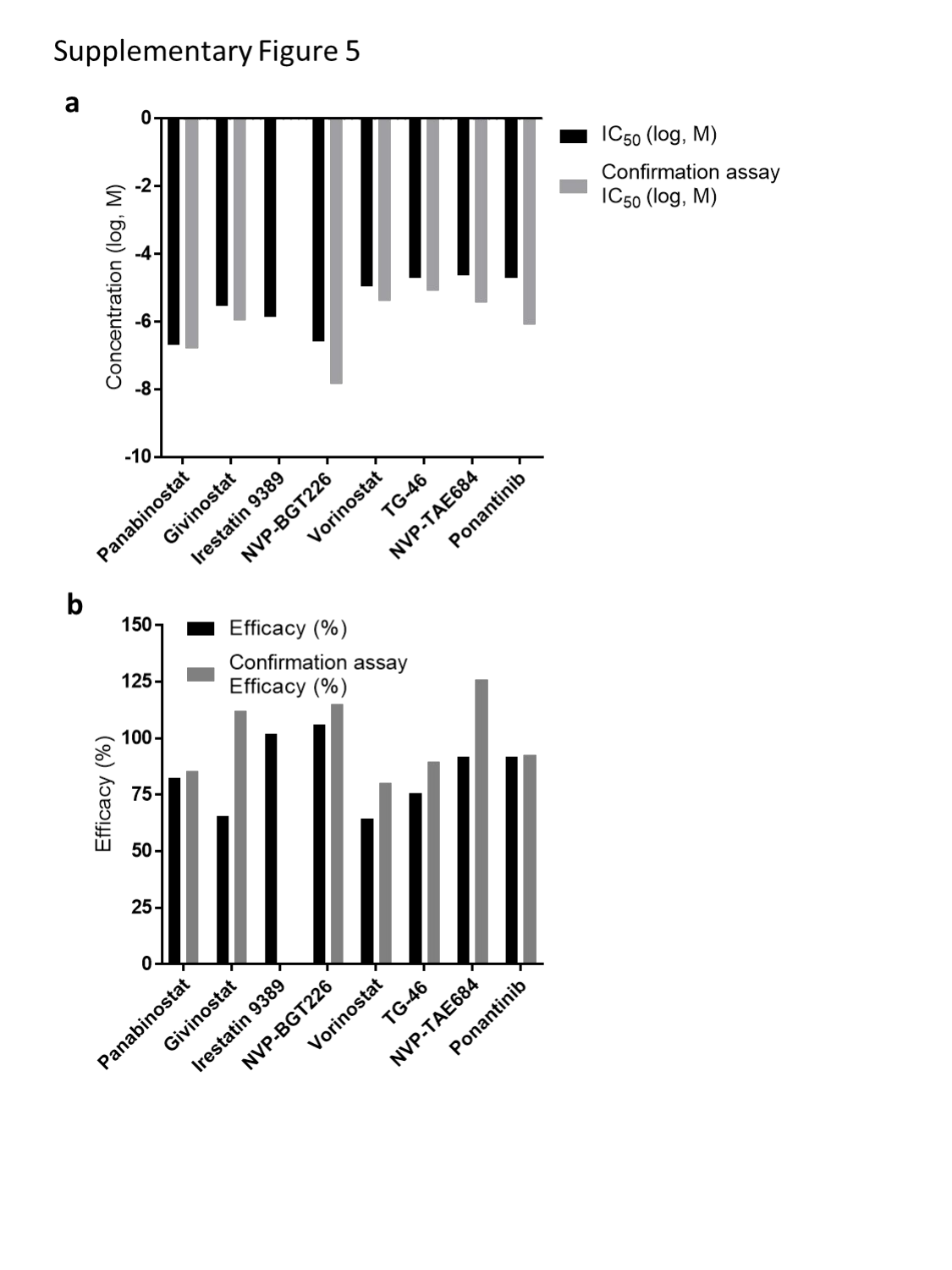

## Slide 6
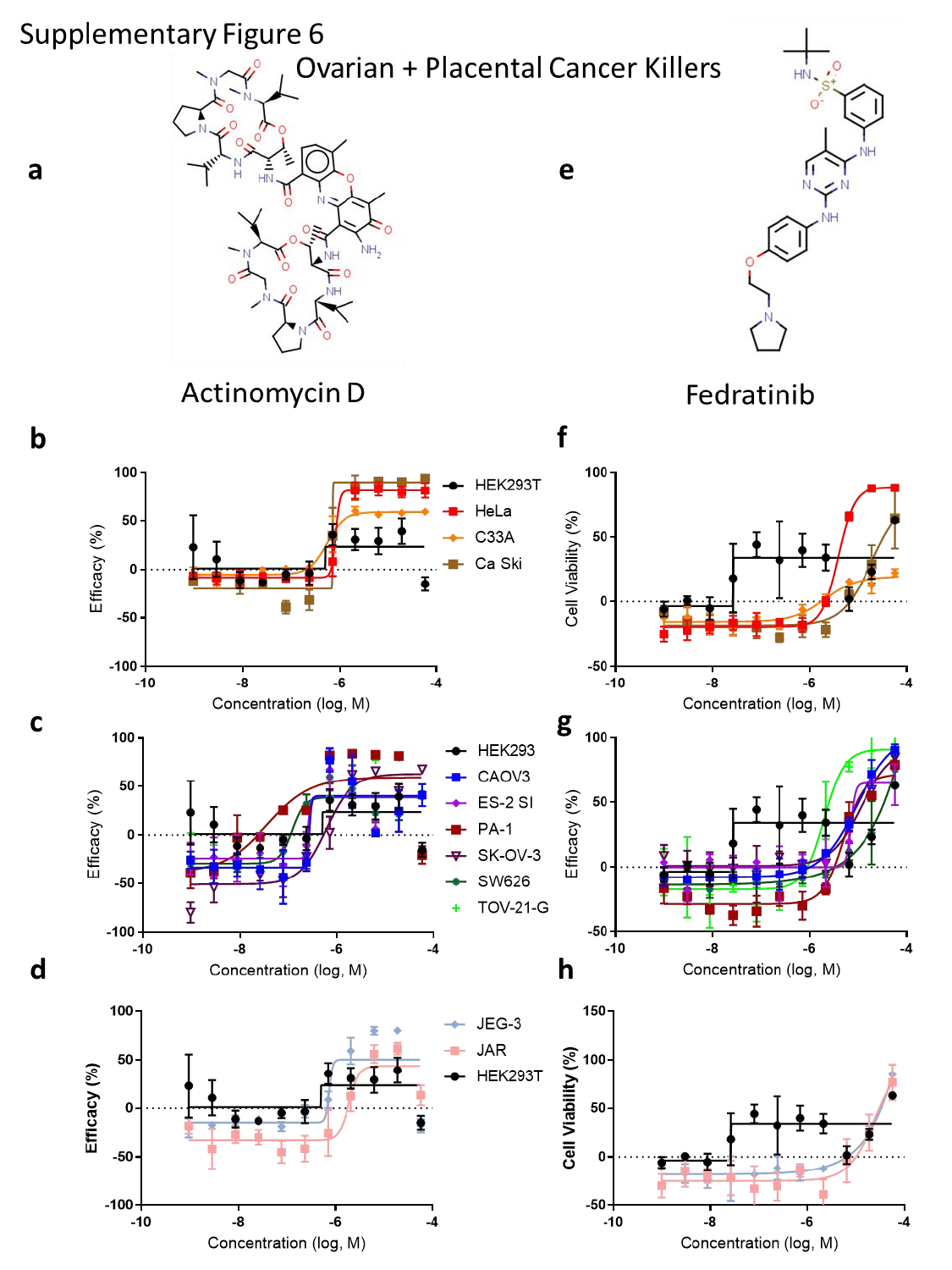

## Slide 7
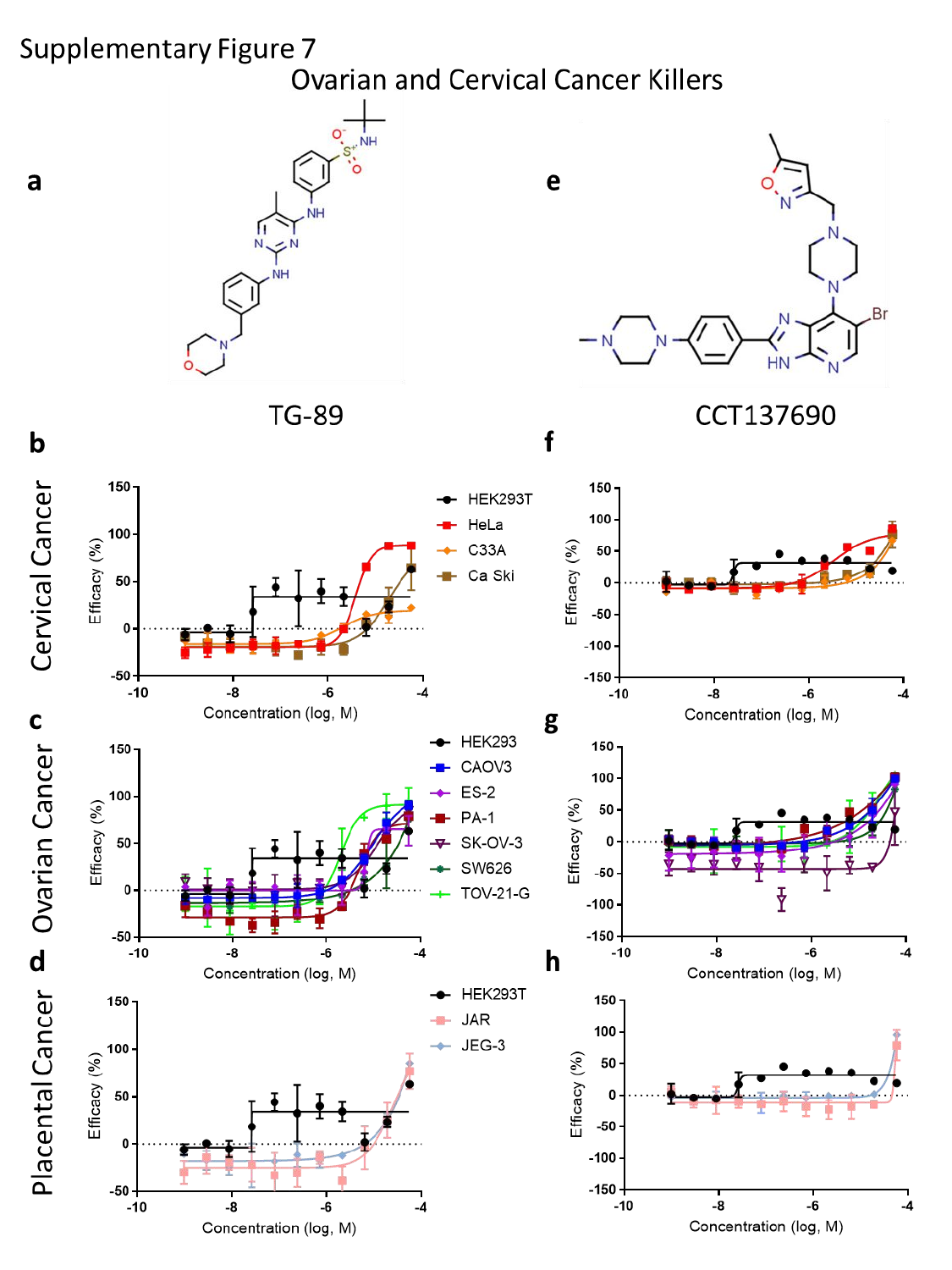

## Slide 8
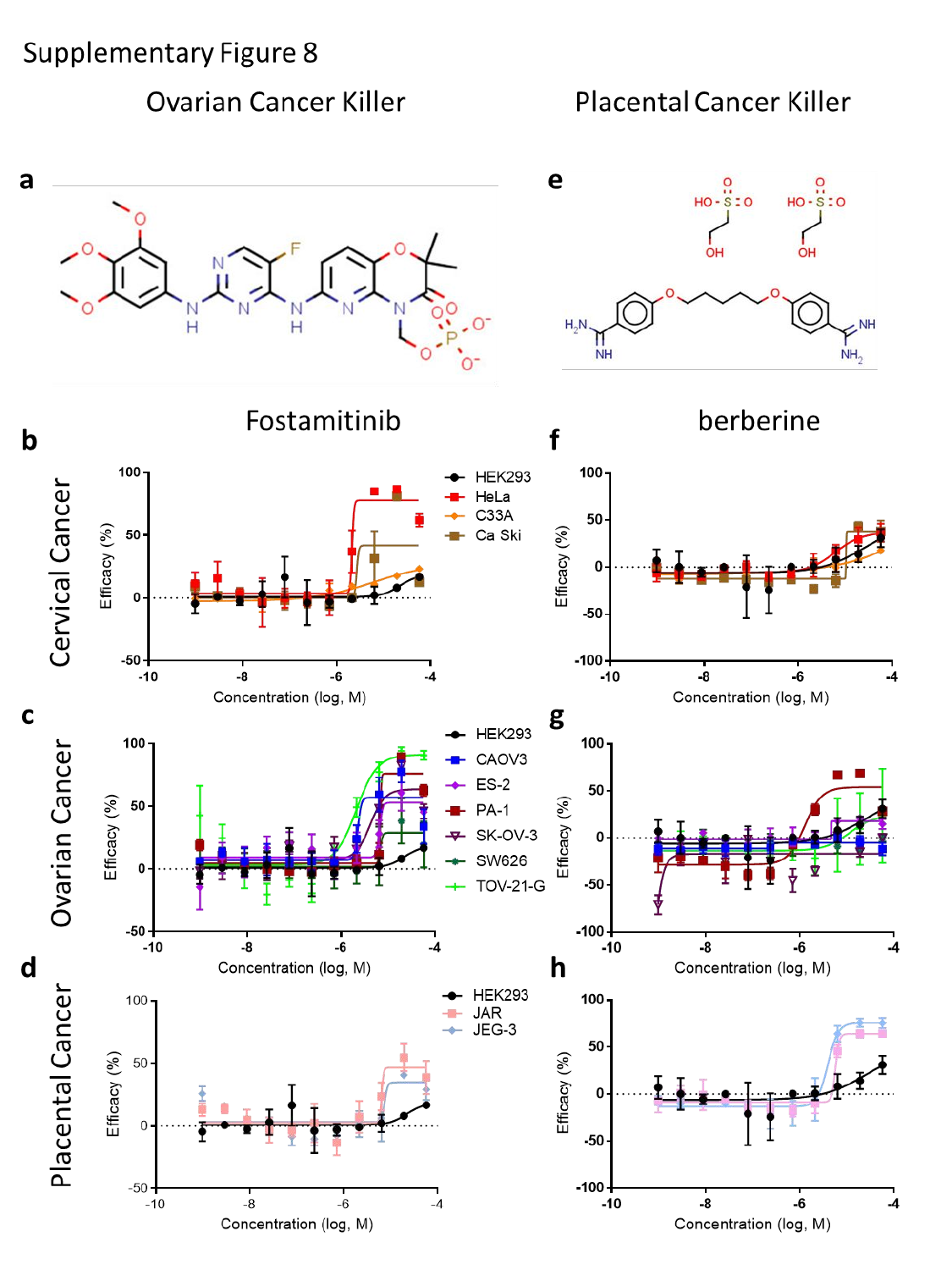

## Slide 9
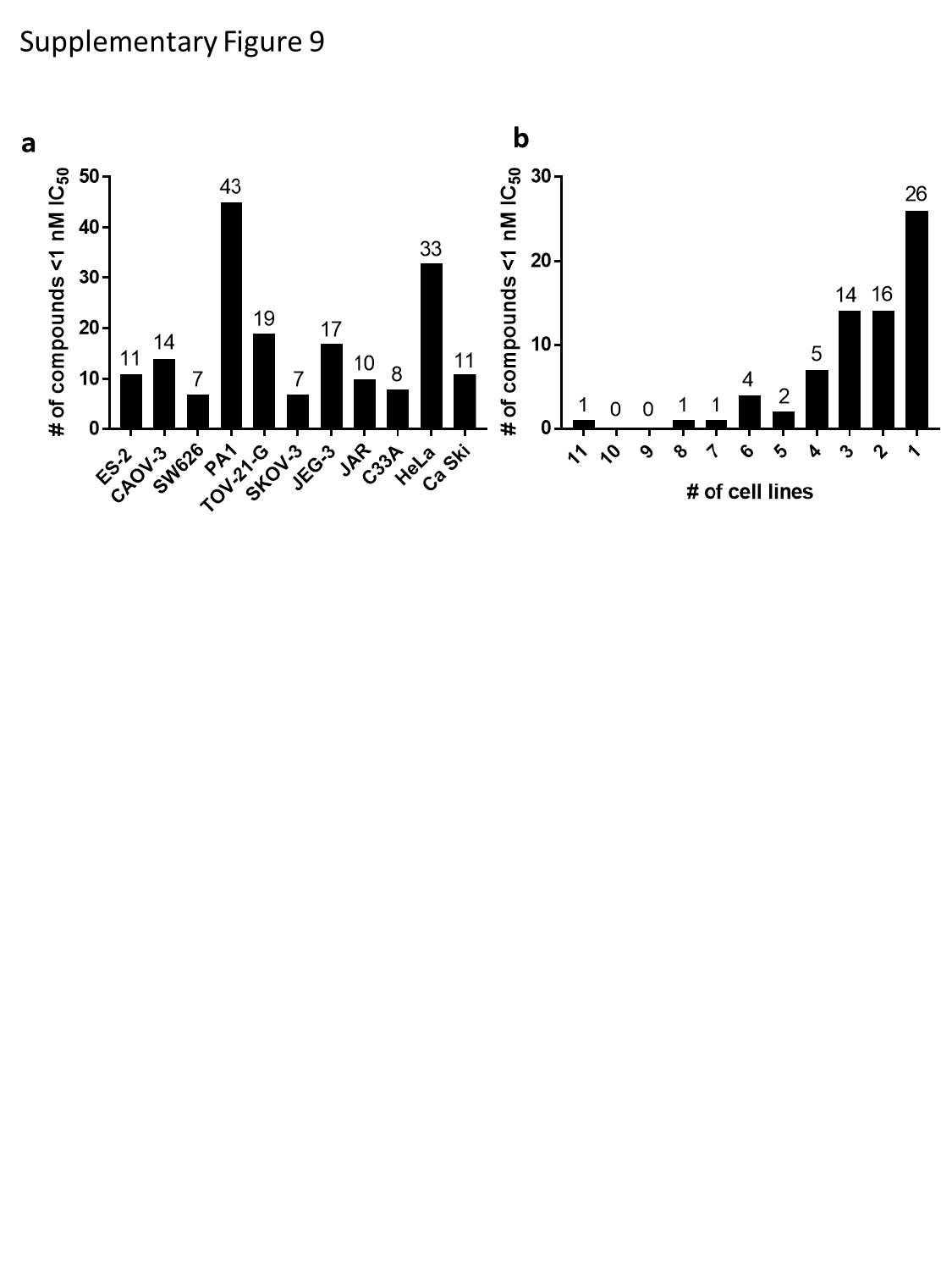

Supplement: Supplementary file 1 — Supplementary Figure 1. Assay development for qHTS screening of chemotherapeutic compounds. ATPlite luminescence from PA-1 ovarian teratocarcinoma cells treated with 10 compounds for 24 (A), 48 (B), and 72 hours (C) in dose-response format including SOC drugs used in the clinic. ATPlite luminescence from CAOV-3 ovarian adenocarcinoma cells treated with 16 compounds for 24 (D), 48 (E), and 72 hours (F) in dose-response format including SOC drugs used in the clinic. Data points representing normalized mean ± S.D. (n=4 wells per data point). Data were normalized to DMSO control (100% cell viability and lowest luminescence value among the 6 compounds (0% cell viability). Curves represent non-linear regression curve fit with variable slope. Supplementary Figure 2. qHTS Assay Workflow for chemotherapeutic profiling of OBGYN cancer cell lines. Supplementary Figure 3. HEK 293T control assay development. ATPlite luminescence from HEK 293T embryonic kidney fibroblasts cells treated with 10 compounds for 24 (A), 48 (B), and 72 hours (C) in dose-response format including SOC drugs used in the clinic. (D) Doxorubicin time course dose-response cell viability curves for PA-1 cells from A, B, and C with IC50 determinations in the inset. (E) Curcubitacin B time course dose-response curves for PA-1 cells from A, B, and C with IC50 determinations in the inset. (F) Log (selectivity index) heat map calculated using the 48-hour time point IC50 values for HEK 293T cells divided by the IC50 values for PA1 and CAOV-3 cells. Table is organized according to the most selective to the least selective compounds. Black panels indicate that no IC50 could be determined from the linear regression curve fit. (G) Chemical structure of doxorubicin. (H) Chemical structure of curcubitacin B. Data points representing normalized mean ± S.D. (n=4 wells per data point). Data were normalized to DMSO control (100% cell viability and lowest luminescence value among the 16 compounds (0% cell viability). Curves r [file mmc1.pptx]
